# Activated gastric cancer-associated fibroblasts contribute to the malignant phenotype and 5-FU resistance via paracrine action in gastric cancer

**DOI:** 10.1186/s12935-018-0599-7

**Published:** 2018-07-20

**Authors:** Yongchen Ma, Jing Zhu, Shanwen Chen, Tengyu Li, Ju Ma, Shihao Guo, Jianwen Hu, Taohua Yue, Junling Zhang, Pengyuan Wang, Xin Wang, Guowei Chen, Yucun Liu

**Affiliations:** Department of General Surgery, Peking University First Hospital, Beijing, 100034 People’s Republic of China

**Keywords:** Gastric cancer, Cancer-associated fibroblasts, Activation, Epithelial–mesenchymal transition, Drug resistance

## Abstract

**Background:**

Cancer-associated fibroblasts (CAFs) play important roles in tumor progression. However, the behaviors of activated CAFs in gastric cancer remain to be determined. The aim of the present study was to investigate the correlations between activated gastric CAFs and the prognosis of patients with gastric cancer, and to determine the effects of activated CAFs on the malignant phenotype and 5-fluorouracil resistance in this cancer.

**Methods:**

Ninety-five patients with primary gastric cancer were enrolled in this study. Activation states of gastric CAFs were evaluated by immunohistochemistry. A modified method for the primary culture of gastric CAFs was employed. Types of CAFs and activation states were identified by immunocytochemical and immunofluorescent staining. Cell co-culture and gastric CAF conditioned medium transfer models were established to investigate the paracrine effects of activated CAFs on the migration and invasion of gastric cell lines. The half maximal inhibitory concentration of 5-fluorouracil and levels of cell apoptosis were examined using cell viability assay and flow cytometry, respectively. Protein expression levels of associated molecules were measured by Western blotting.

**Results:**

Kaplan–Meier survival curves showed that activated gastric CAFs identified via fibroblast activation protein were significantly related to poorer cumulative survival in gastric cancer patients. Five strains of CAFs were successfully cultured via the modified culture method, and three gastric CAFs strains were identified as activated gastric CAFs. The migration and invasion abilities of gastric cells were significantly enhanced in both the co-culture group and the conditioned medium group. The half maximal inhibitory concentration for 5-fluorouracil in BGC-823 cells was elevated after treatment with conditioned medium, and early apoptosis was inhibited. Additionally, an obvious elevation of epithelial–mesenchymal transition level was observed in the conditioned medium group.

**Conclusions:**

Activated gastric CAFs correlate with a poor prognosis of cancer patients and may contribute to the malignant phenotype and the development of resistance to 5-fluorouracil via paracrine action in gastric cancer. Gastric CAFs with a specific activation state might be used as a tumor biomarker within the microenvironment for prognosis and as a new therapeutic target for chemoresistant gastric cancer.

**Electronic supplementary material:**

The online version of this article (10.1186/s12935-018-0599-7) contains supplementary material, which is available to authorized users.

## Background

Since the seed and soil theory was proposed in 2006, accumulating evidence has shown a tight connection between cancer and its microenvironment [1]. The modern cancer model has developed into a cancer-niche complex that includes cancer cells, cancer-associated fibroblasts (CAFs), endothelial cells, immune inflammatory cells, pericytes, and cancer stem cells [2]. Among these components, CAFs, as the major mesenchymal cell component surrounding cancer cells, have various interactions within the featured characteristics of cancer related microenvironment and play a vital role in tumor progression.

Similar to pancreatic stellate cells, CAFs exhibit quiescent and activated phenotypes, and different states contribute diversely to malignant behaviors. In various cancer types, under certain stressful conditions, CAFs can be activated and obtain tumor-promoting functions via proliferation, secretion or metabolic alterations [3–5]. Although previous studies on gastric cancer have introduced the relative behaviors of fibroblasts, most studies to date have not described the identities and states of these fibroblasts [6]. Because of the easily contaminated property of gastric samples and the limitations of primary culture, the functions of gastric CAFs (GCAFs) with specific phenotypes in malignant progression of gastric cancer are poorly understood. Additionally, allopatric fibroblasts are frequently used as substitutes for GCAFs and may be confused with the *bona fide* situation in vivo due to fibroblast heterogeneity.

In this study, we investigated the clinicopathological correlations of activated GCAFs and successfully identified three strains of activated GCAFs from human gastric tumors. Consequently, our in vitro study revealed the possible roles of GCAFs in the malignant phenotype and 5-fluorouracil (5-FU) drug resistance in gastric cancer. Additionally, a modified method of primary culture for GCAFs was also proposed to facilitate further in-depth exploration of targeted treatment based on the tumor microenvironment.

## Methods

### Clinical materials

Ninety-five patients with primary gastric cancer were enrolled in this study, in which 73 patients received tailored follow-up for 5 years (see Additional file 1). Of the cases, 84 patients underwent radical resection of gastric cancer; the remainder (11 cases) received palliative resection. Patients who underwent neoadjuvant treatment, such as chemotherapy or radiotherapy, before surgery were excluded from this study. The pathological diagnosis was confirmed by doctors from the Department of Pathology, Peking University First Hospital, and the classifications of gastric cancer were made based on the AJCC TNM Staging Classification for Carcinoma of the Stomach (7th ed., 2010). Additionally, fresh tumor samples for primary culture were obtained from another three cases in 2017. This study was approved by the Peking University First Hospital Biomedical Research Ethics Committee (No. 2017-37). All patients related to this study signed an informed consent agreement.

### Immunohistochemical analysis

Tumor tissues embedded in paraffin were cut into 3- to 5-µm serial sections and fixed onto slides. EDTA solution (pH 9.0) was applied for antigen retrieval. Following endogenous peroxidase blocking, incubation with rabbit anti-human fibroblast activation protein (FAP) antibody (1:100 dilution; Abcam, MA, USA) and podoplanin (PDPN) antibody (1:250 dilution; CST, MA, USA) were performed overnight. The next day, the sections were incubated with horseradish peroxidase (HRP)-conjugated goat anti-rabbit IgG (ZSGB-BIO, Beijing, China) for 30 min. The DAB staining system was then used to detect the target protein. FAP expression was independently evaluated by three researchers blinded to patient information and outcomes, mainly according to the intensity of staining and scope of the stained region. The semi-quantitative analysis was described by Shi et al. [7]. Briefly, the intensities were scored as follows: 0, no staining; 1, weak staining; 2, intermediate staining; and 3, strong staining. The percentages were scored as follows: 0, complete absence or ≤ 10% staining within the same cell type; 1, 11 to 25%; 2, 26 to 50%; and 3, > 50%. The sum of the scores indicated the expression of FAP: < 3 represented the low-expression group, and ≥ 3 represented the high-expression group.

### Modified method of primary culture for GCAFs

#### Sample sources

Fresh tumor samples were obtained from patients who underwent radical resection of gastric cancer at Peking University First Hospital. Tumor sample #2916 was collected from a patient with adenocarcinoma; #2922 and #2923 were obtained from different focal sites of the same patient with signet ring cell carcinoma. One colon cancer sample and one pancreatic ductal adenocarcinoma sample were also included in this study.

#### Sample dissection

Samples were dissected from the cancer foci during the operation under aseptic conditions. The average size of each sample was 5 × 5 × 5 mm^3^, and 1.5-mL tubes were used to transfer samples, which contained Dulbecco’s Modified Eagle Medium (DMEM, Thermo Fisher Scientific, MA, USA)-high glucose with 10% fetal bovine serum (FBS, Thermo Fisher Scientific, MA, USA), 100 U/mL penicillin and 100 µg/mL streptomycin (Thermo Fisher Scientific, MA, USA), and 200 µg/mL Normocin (InvivoGen, CA, USA). All samples were processed within one hour after dissection.

#### Sample processing

Samples were washed in phosphate-buffered saline (PBS, containing 100 U/mL penicillin, 100 µg/mL streptomycin and 200 µg/mL Normocin) for 30 min to eliminate the most pathogenic microbes. The samples were then cut into 1-mm^3^ pieces without visible adipose tissue. During this procedure, PBS was applied to maintain humidity. Sample pieces were transferred into clean 1.5-mL tubes, and 0.25% trypsin was added to digest the tissues for 30 min.

#### Tissue planting and culture

The mixture of tissues and trypsin digestive medium was transferred into cell culture flasks. The cells were cultured in DMEM with 20% FBS, 100 U/mL penicillin, 100 µg/mL streptomycin, and 200 µg/mL Normocin and incubated in 5% CO_2_ at 37 °C. Additionally, the concentration of FBS was reduced to 5% after the second passage to prevent early senescence.

### Cell lines

The gastric cancer cell lines BGC-823 and SGC-7901 were purchased from the Cancer Institute of the Chinese Academy of Medical Science. MKN-45 cells were obtained from American Type Culture Collection. DMEM-high glucose supplemented with 10% FBS was used to culture BGC-823 and SGC-7901 cells, while RPMI 1640 medium (Thermo Fisher Scientific, MA, USA) was used to culture MKN-45 cells. And cells were cultured in the optimal conditions of 37 °C with 5% CO_2_.

### Immunocytochemical staining

To identify activated GCAFs, we detected four biomarkers at the protein level by immunocytochemical staining. Fibroblasts were seeded on sterilized glass coverslips. After acetone fixation, the coverslips were soaked in 0.75% H_2_O_2_-PBS for 10 min to block endogenous peroxidase. Goat serum was then used as the blocking reagent for 30 min. Antibodies against α-smooth muscle actin (α-SMA; ZSGB-BIO, Beijing, China), vimentin (ZSGB-BIO, Beijing, China), FAP (1:100 dilution), and desmin (ZSGB-BIO, Beijing, China) were incubated with cells individually at 4 °C overnight. HRP-conjugated goat anti-rabbit/mouse IgG antibodies (ZSGB-BIO, Beijing, China) were applied the next day. Next, the cells were counterstained with hematoxylin and sealed with neutral gum.

### Immunofluorescence staining

Immunofluorescence staining was performed on well-cultured fibroblasts. In brief, after fixation, serum blocking and hybridization with primary antibodies overnight, the cells were incubated with fluorescein isothiocyanate (FITC)-conjugated secondary antibodies (1:100 dilution; KeyGEN, Nanjing, China) for 1 h, and the nuclei were stained with 4′,6-diamidino-2-phenylindole (DAPI; ZSGB-BIO, Beijing, China). The cells treated with only secondary antibodies were considered the negative control (NC). The fluorescent expression of the target markers and nuclei were evaluated and imaged using a Leica confocal laser microscope.

### Western blotting

The expression level of proteins in GCAFs and cancer cells were examined as follows. Total cellular proteins were prepared from cell lysates with lysis buffer. As for apoptosis-related proteins detection, cells were treated with 5-FU at the concentration of 1 µg/mL for 72 h with the presence of CM to establish the apoptosis model before protein extraction. After the protein concentration of each sample was adjusted, SDS-polyacrylamide gel electrophoresis was performed to separate proteins. Subsequently, the protein bands were transferred to a polyvinylidene fluoride (PVDF) membrane. The specific primary antibodies were used as follows: FAP (1:1000 dilution), α-SMA (1:10,000 dilution; Abcam, MA, USA), desmin (1:100,000 dilution; Abcam, MA, USA), vimentin (1:1000 dilution), E-cadherin (1:1000 dilution; CST, MA, USA), snail (1:1000 dilution; CST, MA, USA), slug (1:1000 dilution, CST, MA, USA), PARP (1:1000 dilution, CST, MA, USA), cleaved PARP (1:1000 dilution, CST, MA, USA), cleaved caspase 3 (1:1000 dilution, CST, MA, USA), Bak (1:1000 dilution, CST, MA, USA), Bax (1:1000 dilution, CST, MA, USA), tubulin (1:1000, CST, MA, USA) and GAPDH (1:1000, CST, MA, USA). Tubulin and GAPDH served as the internal controls. The level of target proteins were detected using the ECL detection system (Merck, Darmstadt, Germany) and the Syngene GeneGenius gel imaging system (Syngene, Cambridge, UK).

### Wound-healing assay

Gastric cancer cells (BGC-823, MKN-45 and SGC-7901) were co-cultured with GCAFs (cell number 3:1) or in conditioned medium (CM) from GCAFs for 72 h in 6-well plates before the wounds were generated. Cells cultured in DMEM with 5% FBS were used as a control. Next, each well was cultured with serum-free DMEM for 48 h and imaged over this time. The area of the scratched field was measured using ImageJ software, and each sample was assessed in three fields for replicates.

### Transwell invasion assay

The transwell chamber with 8-µm pores was applied in this study to establish the bilayer culture model. The upper chamber was pre-coated with 50 µL of Matrigel (1:8 dilution with DMEM; Corning, NY, USA) and seeded with 1 × 10^5^ BGC-823 cells in serum-free DMEM. GCAFs in complete medium with 5% FBS or in CM from GCAFs were added to the lower chamber. Complete medium alone with 5% FBS was considered the NC. The whole system was cultured for 24 h. Cells penetrating to the lower surface of the transwell chamber were fixed with methanol and stained with crystal violet. Cells were counted in five randomly selected fields for each sample.

### Cell viability assay

The effects of activated GCAFs on the 5-FU resistance of BGC-823 were examined using Cell Counting Kit-8 (CCK-8; Bimake, Shanghai, China). BGC-823 cells pre-cultured in CM from CAF-2916, 2922, and 2923 for 72 h were seeded at 1 × 10^5^/100 µL/well in 96-well plates. Additionally, 5-FU in gradient concentrations from 5 × 10^5^ ng/mL to 5 × 10^−4^ ng/mL (multi-proportion dilution) was added to each well and incubated for 72 h. CCK-8 reagent was applied and incubated for 1.5 h. The absorbance at 450 nm was measured. Wells containing BGC-823 cells in the absence of 5-FU treatment were set as the NC, and wells containing neither BGC-823 cells nor 5-FU treatment were set as the blank control. There were three replicates for each concentration. The dose–effect curves were drawn, and the half maximal inhibitory concentration (IC_50_) of 5-FU was confirmed via multiple linear regression.

### Flow cytometry analysis of apoptosis

Apoptotic cells were detected using the Annexin V-FITC/PI Apoptosis Assay Kit (KeyGen, Nanjing, China) following the manufacturer’s instructions. The group division was the same as that used in the cell viability assay, and the cancer cells were treated with 100 ng/mL of 5-FU for 24 h. After incubation with annexin V-FITC and propidium iodide (PI) for 5 min, the apoptotic status of the cells was analyzed using flow cytometry.

### Statistical analyses

The correlations between the FAP expression and clinicopathology were evaluated using by χ^2^ or Kruskal–Wallis one-way ANOVA, if appropriate. Kaplan–Meier analysis was applied to calculate the survival duration, and the significance between groups was analyzed using the log-rank test. Cox regression analysis was employed to compute multivariate hazard ratios for the study parameters. One-way ANOVA was used for comparison in the wound-healing assay, the transwell assay, and the apoptosis assay. *P *< 0.05 was considered significant, and all tests were analyzed using SPSS 23.0 software.

## Results

### Clinicopathological and survival correlations of activated GCAFs

**Fig. 1:**
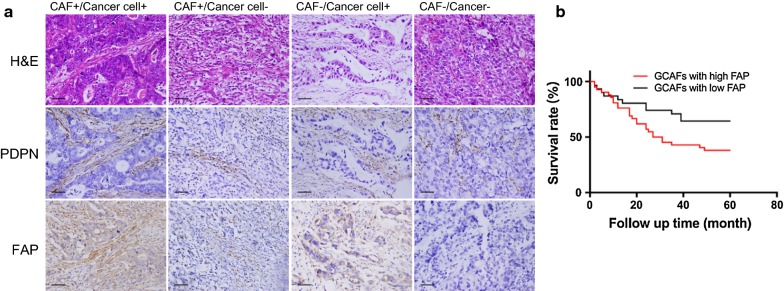
FAP expression and survival of patients with gastric cancer. **a** HE staining and immunohistochemical staining were performed on serial sections of gastric cancer tissues. PDPN expression combined with HE staining was used to trace GCAFs. FAP was mainly located in the cytoplasm in both GCAFs and cancer cells (scale bar = 50 µm). **b** Overall survival of patients (N = 73) was divided into two groups based on FAP expression in GCAFs. The survival curves revealed that the overall survival in gastric cancer patients with high FAP expression in GCAFs was significantly worse than that in patients with low FAP expression (*P *= 0.033)

All cases enrolled in this study were evaluated using immunohistochemistry for tumor tissues. PDPN expression combined with hematoxylin–eosin (HE) staining was used to trace GCAFs, and FAP was employed to indicate the activation state of GCAFs. FAP expression was observed in both GCAFs (61.1%) and cancer cells (86.3%), mainly in the cytoplasm (Fig. 1a). Furthermore, we investigated the correlations between clinicopathology and FAP expression in GCAFs. No significant differences were observed in histological type, grade, tumor location, cancer embolus, or TNM stage (Additional file 2). However, the Kaplan–Meier survival curves revealed a significantly poor overall survival in gastric cancer patients with FAP high expression in GCAFs (*P *= 0.033, Fig. 1b). Multivariate Cox regression analysis showed FAP expression in GCAFs and lymph node metastasis to be independent predictive markers for the survival prognosis of patients (Table 1).

**Table 1:** Multivariate Cox regression analysis for the overall survival of gastric cancer patients

Parameter	B	Wald	*P* value	Hazard ratio	95% CI
FAP expression					
High	0.592	6.596	0.010	2.590	1.253–5.353
Histological type					
Adenocarcinoma	− 0.074	0.027	0.870	0.929	0.384–2.248
Grade					
Moderate to poor	0.456	2.309	0.129	1.578	0.876–2.841
Invasion					
T2–T4	0.363	1.490	0.222	1.437	0.803–2.573
Lymph node metastasis					
Positive	0.615	4.852	0.028	1.849	1.070–3.196
Cancer embolus					
Positive	0.333	0.895	0.344	1.395	0.700–2.780

### Identification of activated GCAFs

**Fig. 2:**
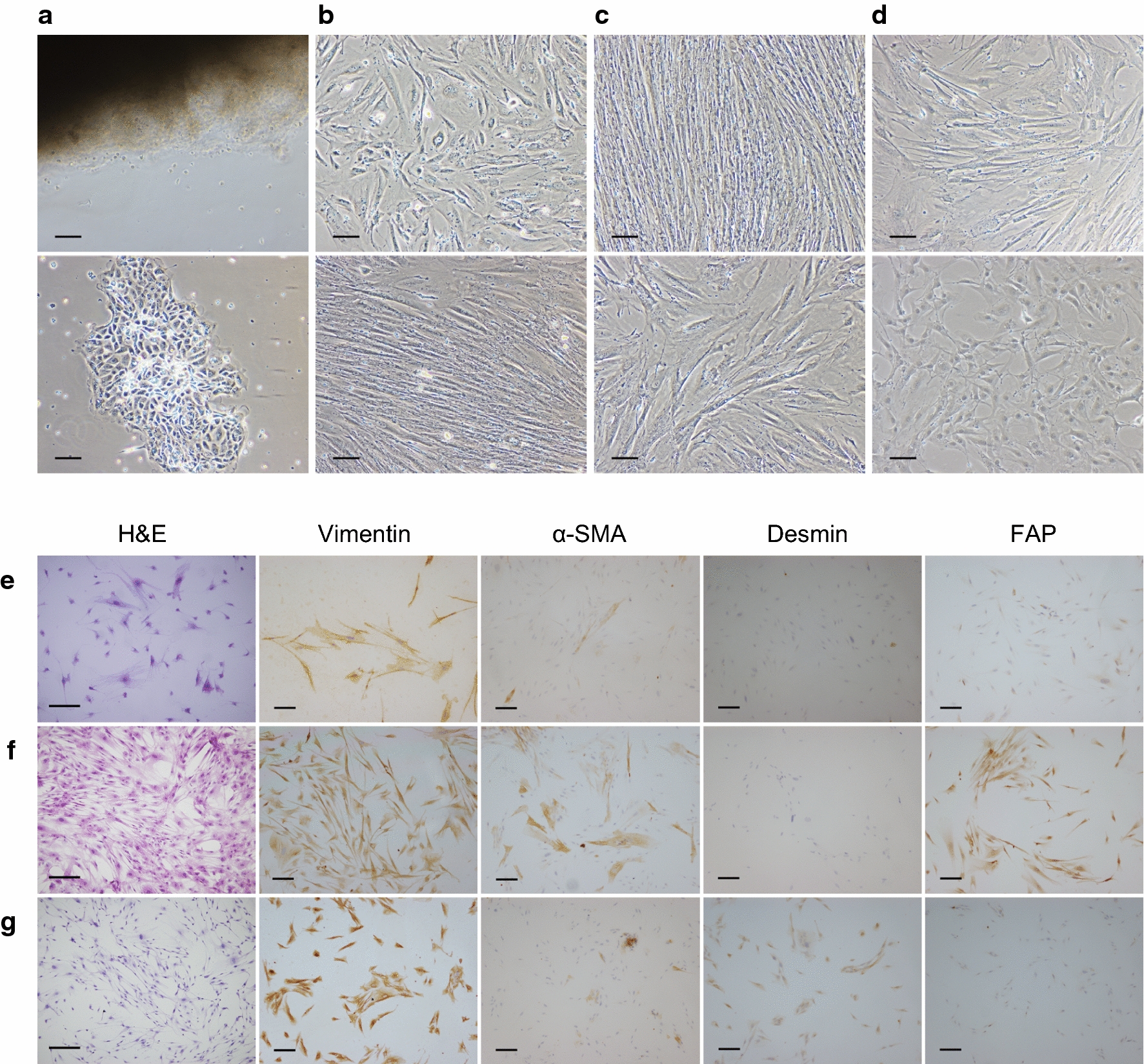
Identification of GCAFs. **a** Characteristics on day 3, floating cells (upper) and an adherent mixture of fibroblasts and cancer cells (lower). **b** Characteristics on day 20, fibroblasts began to show a spindle or a multi-polar morphotype. **c** Characteristics on day 40, three strains of fibroblasts behaved stable, and swirly colonies appeared. **d** Colon (upper) and pancreatic (lower) fibroblasts. H&E staining showed morphological heterogeneity among the three strains of fibroblasts. **e** In CAF-2916, vimentin was expressed at a very high level, and FAP was expressed moderately; α-SMA and desmin were faint. **f** In CAF-2922, vimentin, α-SMA, and FAP were all highly expressed. **g** In CAF-2923, vimentin, desmin, and FAP were found in fibroblasts. Scale bar = 250 µm

Using a modified primary culture method, three strains of GCAFs were successfully cultured and named as follows: CAF-2916, CAF-2922, and CAF-2923. After 3 days, some gastric fibroblasts were adherent, whereas others were floating, and the fibroblasts were mixed with tumor cells (Fig. 2a). The morphologic behaviors varied, and cells were arranged in an unordered mode. After 20 days, the fibroblasts, at the third passage in culture, showed spindle or multi-polar morphotypes (Fig. 2b). Most began to show the same directivity or polarity, and this property became more obvious and stable after 40 days as the confluence increased (Fig. 2c). Additionally, this method was also suitable for colon cancer- and pancreatic cancer-associated fibroblasts, and typical shapes of fibroblasts were observed (Fig. 2d).

**Fig. 3:**
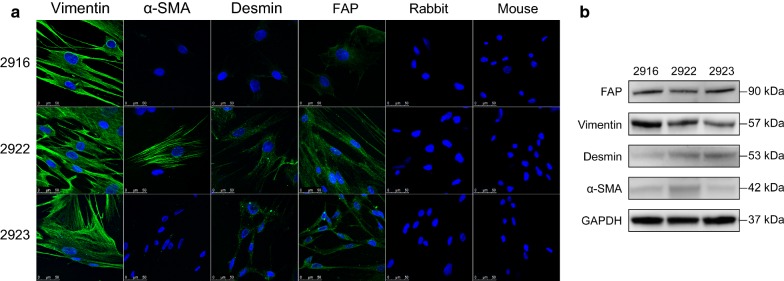
Immunofluorescent staining and semi-quantitative analysis of GCAFs. **a** Proteins for stromal and activation indication were detected by FITC-conjugated secondary antibodies, and the nuclei were stained blue with DAPI. Scale bar = 50 µm. **b** The expression levels were semi-quantitatively detected using western blot analysis. Vimentin and FAP were strongly expressed in all three strains of fibroblasts. α-SMA was relatively high-expressed in CAF-2922, and desmin was low-expressed in CAF-2916

To determine the identity of the primary cells, immunocytochemical staining and immunofluorescent staining were performed. The results of immunocytochemical staining showed that the stromal marker vimentin and activation marker FAP were expressed in the three strains of fibroblasts, though expression of myofibroblastic markers varied. α-SMA was heterogeneously positive in CAF-2922, and desmin was strongly positive in CAF-2923 (Fig. 2e–g). As shown in Fig. 3a, nuclei were stained with DAPI, and the green fluorescence indicated target molecules. Vimentin was strongly expressed in all three fibroblast strains. α-SMA was heterogeneously positive in CAF-2922 but negative in CAF-2916 and CAF-2923. Desmin was detected in CAF-2922 and CAF-2923 but was relatively faint in CAF-2916. FAP was expressed in all three strains. The expression levels were also semi-quantitatively detected using western blot analysis, and the result was consistent with the staining results in general (Fig. 3b). The combination of these molecules recognized the three strains as cancer stromal-derived fibroblasts and helped to identify the activation state of these three GCAFs.

### Activated GCAFs increased the migration abilities of gastric cancer cells

**Fig. 4:**
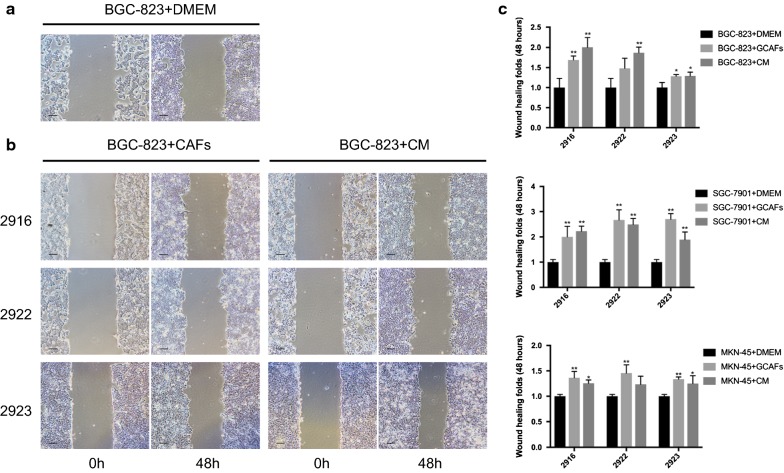
Detection of the migration abilities of gastric cancer cells. BGC-823, MKN-45 and SGC-7901 cells were co-cultured with GCAFs or CM from GCAFs for 72 h before the wounds were generated. Additionally, the scratch area after 48 h was measured to evaluate the migration abilities of cancer cells (scale bar = 500 µm). **a** Cells cultured in DMEM with 5% FBS were used as the control. **b** Alterations of BGC-823 cells treated with CAF-2916, CAF-2922, and CAF-2923. **c** Ratios of the migration area of gastric cancer cells (BGC-823, MKN-45 and SGC-7901) treated with activated GCAFs compared with those of the control group after 48 h (normalized). *P < 0.05, **P < 0.01

Wound-healing assays were performed on BGC-823, MKN-45 and SGC-7901 cell lines. The results showed that the scratch areas in the co-culture group and the CM group were both smaller than those in the DMEM control group after 48 h of culture. In the CAF-2916 group, BGC-823 cells were significantly accelerated in migration compared with that in the control group, and the wound-healing area in the co-culture group was 1.689 ± 0.100-fold higher than that in the DMEM control group (*P *< 0.01). Furthermore, BGC-823 cells cultured with CM from CAF-2916 also showed a promotion of migration potential (2.005 ± 0.239-fold, *P *< 0.01) (Fig. 4 and Additional file 3). The three strains of activated GCAFs increased the migration abilities of the three gastric cancer cell lines in varying degrees.

### Activated GCAFs increased the invasion abilities of gastric cancer cells

**Fig. 5:**
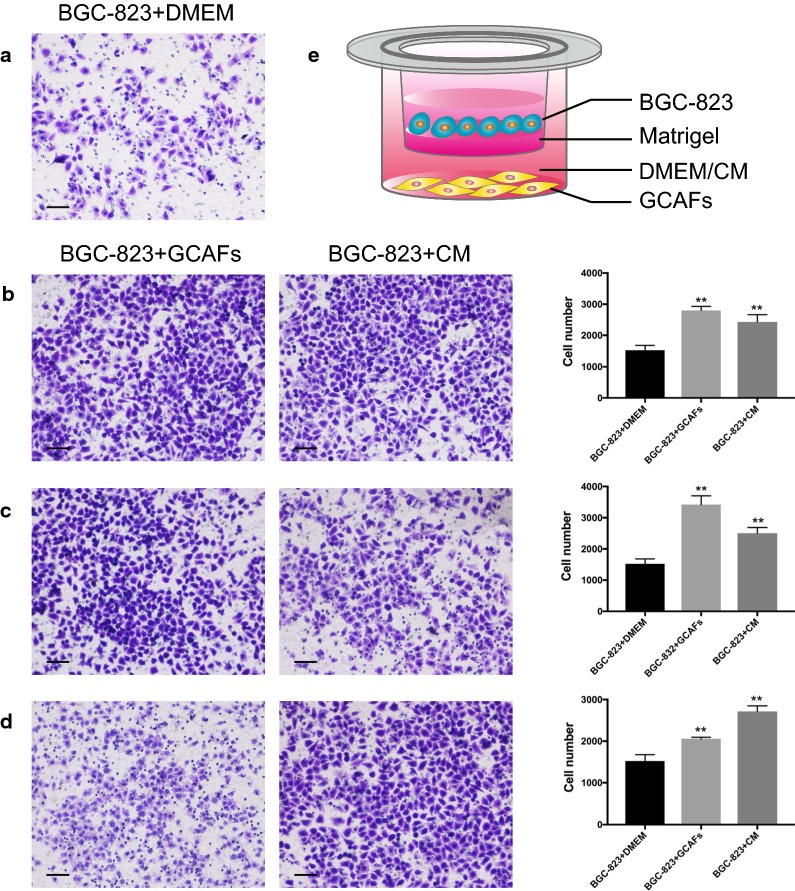
Detection of the invasion abilities of gastric cancer cells. **a** Cells cultured in DMEM with 5% FBS were used as the control. **b**–**d** Images of invading BGC-823 cells and the comparisons between the GCAF-/CM- treated groups and DMEM control group (scale bar = 250 µm). **e** Bilayer culture model. The upper chamber was seeded with BGC-823 cells, and the lower chamber was seeded with or without GCAFs/CM. **P < 0.01

The bilayer culture model was successfully established to observe the possible functions of activated GCAFs in cancer invasion (Fig. 5e). The transwell invasion assay revealed increased invasion abilities of BGC-823 cells treated with the three strains of activated GCAFs (F = 8.086, *P *= 0.020). After co-culture with CAF-2916 for 72 h, the number of BGC-823 cells penetrating to the lower surface of the transwell chamber was significantly increased compared with the control group (2800.000 ± 129.677 vs. 1522.000 ± 155.319, respectively). Additionally, the cell number in the CM group was 2430.667 ± 233.791, which was also significantly increased compared with the control group (Fig. 5a–d).

### Activated GCAFs decreased the 5-FU response of gastric cancer cells

**Fig. 6:**
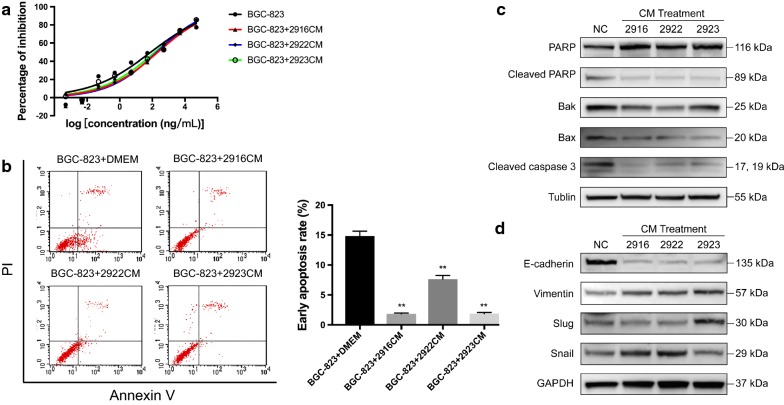
5-FU drug-resistance assays. **a** The dose–effect curves were drawn based on the CCK-8 assay. All curves presented a right shift in the CM group compared with the control group. **b** Apoptosis analysis. After treatment with 5-FU (100 ng/mL) for 24 h, more apoptotic cells (especially in the early stage) were observed in all three CM-treated groups. **c** Expression level of PARP, cleaved PARP, Bak, Bax and cleaved caspase 3, after treatment with 5-FU for 72 h. **d** Expression level of E-cadherin, vimentin, snail and slug after treatment with CM for 72 h

To explore the contributions of paracrine factors from activated GCAFs to the drug response, BGC-823 cells were cultured in CM, and the CCK-8 assay was performed to examine cancer cell viability. The dose–effect curves presented a shift to the right in the CM group compared with that in the DMEM control group (Fig. 6a). After a 72-hour incubation in CM from three strains of activated GCAFs, the IC_50_ values of 5-FU in BGC-823 cells were elevated as follows: CAF-2916, 219.2 ng/mL, 95% confidence interval (CI) 102.1–485.6 ng/mL; CAF-2922, 186.6 ng/mL, 95% CI 107.9–325 ng/mL; CAF-2923, 145.2 ng/mL, 95% CI 56.48–383.2 ng/mL; and the DMEM control group 80.55 ng/mL, 95% CI 26.1–266.3 ng/mL. In the flow cytometry apoptosis analysis, decreases in the proportion of early apoptotic cells were observed in all the CM-treated groups compared with those in the control group (F = 421.3, *P *< 0.0001) (Fig. 6b). Western blotting analysis showed that after treatment with 5-FU, the expression levels of Bak, Bax, cleaved caspase 3 and cleaved PARP in the CM-treated group were all downregulated compared with the control group (Fig. 6c). CM from the three strains of GCAFs inhibited apoptosis of cancer cells.

### Activated GCAFs promoted the epithelial–mesenchymal transition (EMT) of gastric cancer cells

The expression of E-cadherin, vimentin and EMT-related transcription factors snail and slug was tested with Western blotting in BGC-823 cells after treatment with CM from three strains of activated GCAFs. The results suggested that CM promoted the expression of vimentin and inhibited the expression of E-cadherin compared with the DMEM control group. Additionally, snail was upregulated in the CAF-2916 group and the CAF-2922 group, while slug was upregulated in the CAF-2923 group (Fig. 6d).

## Discussion

A plethora of studies have revealed the roles of the microenvironment in neoplasms. As the key component with the largest proportion, CAFs are reported to participate in tumor-stroma crosstalk and to exhibit similarities as well as peculiarities in different solid tumors [8–10]. Because of the difficulty in establishing primary GCAF cultures, other CAFs not from the stomach have been frequently applied in several gastric cancer studies, which likely does not sufficiently mimic the *bona fide* microenvironment in gastric cancer [11]. Therefore, The behaviors of activated GCAFs in gastric cancer remain poorly understood.

In this study, to investigate the roles of activated GCAFs in gastric cancer patients, we used HE staining combined with PDPN detection to trace GCAFs, and FAP was employed to indicate the activation state of GCAFs. PDPN is a widely-accepted marker of CAFs, which was highly expressed in gastric fibroblasts but absent in cancer cells [12, 13]. FAP, initially known as F19 antigen, is an integral membrane serine protease that is highly expressed in activated fibroblasts during tissue rebuilding. In recent years, increasing evidence has showen that FAP is present in several CAFs. Additionally, high expression is detected in the tumor microenvironment, but FAP is rarely detectable in normal tissues [14]. Jochen et al. [15] found that FAP expression indicated the activation state of fibroblasts, which was helpful to identify the activated CAFs. A significant correlation between FAP and the poor 5-year survival was observed in this study. However, no certain clinicopathological relevance of FAP-identified GCAFs was found among the 95 cases. A meta-analysis including 15 studies concerning FAP expression in the stroma cells of several solid tumors also drew a negative conclusion, implying other underlying ways in which GCAFs influence prognosis [16].

Because patients with gastric cancer often develop chronic atrophic gastritis, the reduction of gastric acid causes the mucosa to be contaminated easily by microorganisms, contributing to the failure of primary culture [17, 18]. Although immortalized stromal cell lines have been established for study by some institutes, the primary culture of gastric CAFs should provide more convincing results for experiments in vitro. A modified method for the primary culture of GCAFs was proposed in the present study. To reduce the chance of contamination, the position below the mucosal surface was suggested as the optimal site for sampling in our study. Normocin has shown to effectively prevent mycoplasma, bacterial and fungal contamination [19]. Our experience was that, at the early stage of primary culture, the Normocin concentration should be twice as high as the recommended dose (100 µg/mL), and a combination of Normocin with penicillin–streptomycin solutions may broaden the anti-bacterial spectrum. After sample dissection, washing tissues in PBS containing a high concentration of antibiotics for at least 30 min was the key to reduce contamination in the following steps. In the pre-experimental period, one case treated with only normal penicillin–streptomycin solutions was polluted by fungi. The addition of Normocin (200 µg/mL) guaranteed sample quality.

Traditional methods for primary culture mainly include enzymatic digestion and tissue planting [20, 21]. Here, we combined the two methods. The mixture of digested cells together with tissue blocks was planted into flasks to improve the cell-planting rate and shorten the culture cycle. To avoid possible cell disruption, filtration through a nylon mesh is not recommended.

Vimentin is a 57-kDa cytoplasmic protein, which is one of the most widespread intermediate filament proteins expressed in almost all mesenchymal cells. α-SMA expression usually appears in the transformation of fibroblasts to myofibroblasts around cancer cells, which could modulate the malignant cancer phenotypes. CAFs with the myofibrobalstic phenotype usually undergo autophagy at a relative high level, which partially accounts for chemoresistance of cancer [22]. Additionally, this biomarker also helps to indicate CAF activation [11, 23, 24]. Desmin is expressed in smooth muscle cells, myocardial cells, skeletal muscle cells and fibroblasts. The combination of these molecules could help to recognize the cancer stromal-derived fibroblasts and identify the activation state of CAFs.

Based on the results of immunocytochemical and immunofluorescent staining, vimentin was expressed in all three strains of fibroblasts, and the expression of α-SMA and desmin varied among the three strains, indicating the stromal origin and differentiation potential of the primary cultured cells. Furthermore, FAP was detected to demonstrate the activation state. Given the morphological behaviors and vigorous ability of proliferation, these three strains of GCAFs were defined as activated GCAFs.

The well-cultured activated GCAFs were subsequently applied in the functional experiments in our study. The results from wound-healing and transwell invasion assays revealed the increasing capacities of migration and invasion by gastric cancer cells in vitro after treatment with CM or co-culture with GCAFs. This consistency indicated that CM might contain promoting factors secreted by GCAFs, and these factors contributed to the malignant phenotype. Moreover, an obvious elevation of EMT level was also observed in the CM treated-groups, and different GCAFs triggered distinct transcriptional pathways. The upregulation of EMT-related transcription factors snail and slug demonstrates the EMT progression is promoted in the transcription level. E-cadherin participates in the regulation of adhesions between cells, and low expression indicates the increased invasiveness of cancer cells. In this study, CAF-2922 and CAF-2923 were obtained from different focal sites of the same patient with signet ring cell carcinoma. However, the molecular expression and biological features were different, which indicated the heterogeneity of activated GCAFs even in the same individual.

The supporting and promoting effects of CAFs on malignancy have been reported in several solid tumors, in which paracrine action played an important role. Hwang et al. [25] found that CM from human pancreatic stellate cells could stimulate the proliferation, migration, invasion and colony formation of pancreatic cancer cells dose-dependently, and soluble factors in CM may contribute to these phenomena via activation of the MAPK and AKT pathways in tumor cells. Human mammary epithelial cells acquire the mesenchymal phenotype when co-cultured with CAFs, and an increase in phosphorylated Smad2, Erk1/2, and Jun is observed [26]. Based on the reverse Warburg effect theory, some studies indicated that caveolin1-null CAFs could perform both aerobic glycolysis and autophagy to provide energy substance for the neighboring cancer cells, and this metabolic symbiosis also contributes to the malignant phenotypes of cancer [27]. All of these findings suggest that in the stomach, multiple factors and signaling pathways might be involved in the effects of activated GCAFs in promoting the malignant phenotype.

The failure of chemotherapy is a great dilemma for the long-term survival of gastric cancer patients. 5-FU, the most widespread antimetabolite in cancer chemotherapy, can attenuate DNA synthesis via the inhibition of thymidylate synthetase in gastric cancer cells. In this study, we collected GCAF-conditioned growth medium (7 days) to cultivate BGC-823 cells to explore the role of activated GCAFs in the response to 5-FU in gastric cancer cells. The dose–effect curves of the CM group presented a shift toward the right, and the IC_50_ values of 5-FU in BGC-823 cells were all significantly elevated. The 5-FU response of gastric cancer was decreased after treatment with CM from activated GCAFs, and the serial downregulation of apoptosis-related proteins was observed, which might indicate an anti-mitochondrial pathway apoptosis effect from activated GCAFs via paracrine action [28]. In previous studies, the mechanisms of drug resistance have mainly focused on cancer cells themselves, whereas the involvement of the tumor microenvironment has only recently been recognized [29, 30].

Some findings have revealed that gemcitabine, another common chemotherapeutic drug, could be trapped within CAFs, making the drug unavailable [31]. Not only CAFs themselves but also the factors they secrete participate in the modulation of chemoresistance. Hepatocyte growth factor (HGF) secreted by fibroblasts leads to the resistance of BRAF-mutant melanoma to RAF inhibition via activation of the MAPK pathway, the PI3 K/AKT pathway, and the HGF receptor MET, while similar phenomena also appear in colorectal cancer [32–34]. Based on the expression of invasive marks in other cancer types, we have checked some potential biomarkers in GCAFs from the Human Protein Atlas. As a result, HGF, platelet derived growth factor A and WNT16, reported to be produced by CAFs from other cancer types, are also expressed in GCAFs, which correlate with the poor overall survival [35]. However, these CAF theories have also been challenged. Geller et al. [36] found that drug resistance in pancreatic ductal adenocarcinoma was induced by intratumor *M. hyorhinis* and not by fibroblasts, and the response was recovered after killing the microbes or filtering the CM. In our study, Normocin proved to eliminate mycoplasmas effectively when combined with penicillin–streptomycin, which were applied in the culture of GCAFs, and a significant decrease in the 5-FU response was still observed in the CCK-8 assay. For gastric cancer, activated GCAFs should be a candidate to account for the drug response in the tumor microenvironment.

## Conclusions

Activated GCAFs can promote migration as well as invasion and contribute to 5-FU resistance in gastric cancer cells via paracrine action, indicating that activated GCAFs may serve as a promising prognosis marker of gastric cancer and as a therapeutic target for chemoresistance. Additionally, a modified method for the primary culture of GCAFs was developed and might facilitate future investigation of the detailed mechanisms in the tumor microenvironment.

## Additional files

**Additional file 1.** Information data of the cases enrolled in this study. This file includes the clinicopathological information of cases enrolled in this study.

**Additional file 2.** Correlations between FAP staining and the clinicopathology of gastric cancer. The table in this file shows the correlations between FAP staining in GCAFs and the clinicopathology of gastric cancer.

**Additional file 3.** Wound-healing assay in MKN-45 and SGC-7901 cell lines. MKN-45 and SGC-7901 cells were co-cultured with GCAFs or CM from GCAFs for 72 hours before the wounds were generated. And the scratch area after 48 hours was measured to evaluate the migration abilities of cancer cells (scale bar=500 µm).

## Abbreviations

CAFs: cancer-associated fibroblasts
GCAFs: gastric cancer-associated fibroblasts
5-FU: 5-fluorouracil
FAP: fibroblast activation protein
PDPN: podoplanin
HRP: horseradish peroxidase
DMEM: Dulbecco’s Modified Eagle Medium
FBS: fetal bovine serum
PBS: phosphate-buffered saline
α-SMA: α-smooth muscle actin
FITC: fluorescein isothiocyanate
DAPI: 4′,6-diamidino-2-phenylindole
NC: negative control
PVDF: polyvinylidene fluoride
CM: conditioned medium
CCK-8: Cell Counting Kit-8
IC_50_: half maximal inhibitory concentration
PI: propidium iodide
HE: hematoxylin–eosin
EMT: epithelial–mesenchymal transition
CI: confidence interval
HGF: hepatocyte growth factor
