# Supplementary material for: Activated gastric cancer-associated fibroblasts contribute to the malignant phenotype and 5-FU resistance via paracrine action in gastric cancer
Source: Cancer Cell Int. 2018 Jul 20;18:104. doi: 10.1186/s12935-018-0599-7 (PMC6053778; doi:10.1186/s12935-018-0599-7)
Supplement: Supplementary file 2 — Additional file 2. Correlations between FAP staining and the clinicopathology of gastric cancer. The table in this file shows the correlations between FAP staining in GCAFs and the clinicopathology of gastric cancer. [file 12935_2018_599_MOESM2_ESM.docx]

**Additional file 2** Correlations between FAP staining and clinicopathology of gastric cancer

| Parameter | No. of cases | FAP expression | | *P*-value |
| --- | --- | --- | --- | --- |
|  |  | High | Low |  |
| Gender | 95 |  |  |  |
| Female | 17 | 9 (52.9%) | 8 (47.1%) | 0.449 |
| Male | 78 | 49 (62.8%) | 29 (37.2%) |  |
| Age | 95 |  |  |  |
| ＜70 | 65 | 40 (61.5%) | 25 (38.5%) | 0.886 |
| ≥70 | 30 | 18 (60.0%) | 12 (40.0%) |  |
| Histological type | 95 |  |  |  |
| Adenocarcinoma | 73 | 43 (58.9%) | 30 (41.1%) | 0.434 |
| Other type | 22 | 15 (68.2%) | 7 (31.8%) |  |
| Grade | 88 |  |  |  |
| Well differentiated | 12 | 8 (66.7%) | 4 (33.3%) | 0.402 |
| Moderately differentiated | 34 | 21 (61.8%) | 13 (38.2%) |  |
| Poorly differentiated | 42 | 23 (54.8%) | 19 (45.2%) |  |
| Tumor location | 95 |  |  |  |
| Fundus-cardia | 21 | 13 (61.9%) | 8 (38.1%) | 0.849 |
| Body | 26 | 14 (53.8%) | 12 (46.2%) |  |
| Antrum | 40 | 26 (65.0%) | 14 (35.0%) |  |
| Diffused | 8 | 5 (62.5%) | 3 (37.5%) |  |
| Degree of invasion | 95 |  |  |  |
| T1 | 3 | 2 (66.7%) | 1 (33.3%) | 0.457 |
| T2 | 18 | 10 (55.6%) | 8 (44.4%) |  |
| T3 | 46 | 32 (69.6%) | 14 (30.4%) |  |
| T4 | 28 | 14 (50.0%) | 14 (50.0%) |  |
| Lymph node metastasis | 95 |  |  |  |
| 0 | 20 | 14 (70%) | 6 (30%) | 0.848 |
| 1-2 | 14 | 5 (35.7%) | 9 (64.3%) |  |
| 3-6 | 26 | 17 (65.3%) | 9 (34.7%) |  |
| 7 or more | 35 | 22 (62.9%) | 13 (37.1%) |  |
| Cancer embolus | 95 |  |  |  |
| Yes | 42 | 27 (64.3%) | 15 (35.7%) | 0.565 |
| No | 53 | 31 (58.5%) | 22 (41.5%) |  |
| Stage | 95 |  |  |  |
| I-II | 34 | 21 (61.8%) | 13 (38.2%) | 0.915 |
| III | 61 | 37 (60.7%) | 24 (39.3%) |  |
