# Supplementary figures and images for: Activated gastric cancer-associated fibroblasts contribute to the malignant phenotype and 5-FU resistance via paracrine action in gastric cancer
Source: Cancer Cell Int. 2018 Jul 20;18:104. doi: 10.1186/s12935-018-0599-7 (PMC6053778; doi:10.1186/s12935-018-0599-7)

SGC-7901+DMEM

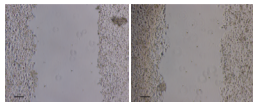

MKN-45+DMEM

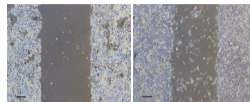

SGC-7901+CAFs

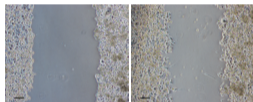

SGC-7901+CM

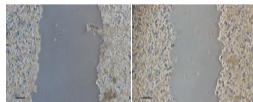

MKN-45+CAFs

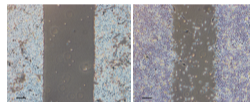

MKN-45+CM

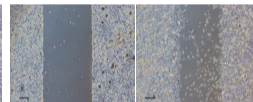

2916

2916

2922

2922

2923

2923

0h

48h

0h

48h

0h

48h

0h

48h

Supplement: Supplementary file 3 — Additional file 3. Wound-healing assay in MKN-45 and SGC-7901 cell lines. MKN-45 and SGC-7901 cells were co-cultured with GCAFs or CM from GCAFs for 72 hours before the wounds were generated. And the scratch area after 48 hours was measured to evaluate the migration abilities of cancer cells (scale bar=500 µm). [file 12935_2018_599_MOESM3_ESM.pdf]
